# Adjuvant Strategies for More Effective Tuberculosis Vaccine Immunity

**DOI:** 10.3390/microorganisms7080255

**Published:** 2019-08-12

**Authors:** Erica Stewart, James A Triccas, Nikolai Petrovsky

**Affiliations:** 1 Discipline of Infectious Diseases and Immunology, Central Clinical School, Faculty of Medicine and Health, The University of Sydney, Camperdown, NSW 2006, Australia; 2 Charles Perkins Centre, The University of Sydney, Camperdown, NSW 2006, Australia; 3 Vaxine Pty Ltd., Adelaide, SA 5042, Australia; 4 Department of Endocrinology, Flinders University, Adelaide, SA 5042, Australia

**Keywords:** Tuberculosis, vaccine, adjuvant, delta inulin, mucosal, immunology, clinical trials

## Abstract

Tuberculosis (TB) caused by *Mycobacterium tuberculosis* infection is responsible for the most deaths by a single infectious agent worldwide, with 1.6 million deaths in 2017 alone. The World Health Organization, through its “End TB” strategy, aims to reduce TB deaths by 95% by 2035. In order to reach this goal, a more effective vaccine than the Bacillus Calmette-Guerin (BCG) vaccine currently in use is needed. Subunit TB vaccines are ideal candidates, because they can be used as booster vaccinations for individuals who have already received BCG and would also be safer for use in immunocompromised individuals in whom BCG is contraindicated. However, subunit TB vaccines will almost certainly require formulation with a potent adjuvant. As the correlates of vaccine protection against TB are currently unclear, there are a variety of adjuvants currently being used in TB vaccines in preclinical and clinical development. This review describes the various adjuvants in use in TB vaccines, their effectiveness, and their proposed mechanisms of action. Notably, adjuvants with less inflammatory and reactogenic profiles that can be administered safely via mucosal routes, may have the biggest impact on future directions in TB vaccine design.

## 1. Introduction

Tuberculosis (TB) is responsible for the most deaths by a single infectious agent worldwide, with 1.6 million deaths in 2017 alone [1]. The causative agent, *Mycobacterium tuberculosis*, is a slow growing organism that is equipped with many immune evasion strategies, making it difficult to treat. The vaccine currently in use, Bacillus Calmette-Guerin (BCG), protects children against disseminated TB and meningitis, but provides incomplete and variable protection against pulmonary TB, the most common form of the disease [2]. The World Health Organization through its “End TB” strategy, aims to reduce TB deaths by 95% by 2035, and to reach this goal, a more effective vaccine will need to be developed.

The current pipeline of TB vaccine candidates is highly varied and includes live, whole cell inactivated, viral vector and subunit vaccines [3,4]. A new TB vaccine will ideally work as an effective booster to BCG vaccination, as it is unlikely that BCG will be completely replaced in many countries, given its efficacy against severe childhood forms of TB and its apparent ability to reduce pediatric infectious disease deaths more generally, through nonspecific mechanisms [5,6]. Ideally, a new TB vaccine would also be suitable as both a pre-exposure preventative and therapeutic post-exposure vaccine, the latter boosting the existing immunity in order to control ongoing infection or preventing the reactivation of latent TB. As *M. tuberculosis* infection is a major cause of death in HIV infected individuals, ideally the vaccine should be suitable for immunocompromised individuals for whom BCG is contraindicated [2]. 

The vaccine strategies most advanced in the TB vaccine clinical pipeline are killed whole-cell bacterial formulations, namely *M. vaccae* and *M. indicus pranii*, which are currently undergoing phase III trials [7]. Antigen expression dynamics may be crucial for generating effective immunity and whole-cell killed vaccines, and while they express many antigens, they may nevertheless not express enough of the right antigens for robust protection [8]. Live vaccines, such as viral vectored, modified BCG, and attenuated *M. tuberculosis*, are also being trialed. Live vaccines have the advantage of a sustained antigen expression, and non-replicating vectors such as adenovirus may be safe for use in HIV-infected individuals [9]. However, while the use of viral vectored vaccines often promotes strong Th1 responses, which, as exemplified by the outcomes of the phase IIb MVA85A trial, does not necessarily correlate with protection against *M. tuberculosis* [10].

Subunit approaches provide flexible design, allowing for the targeting of all stages of *M. tuberculosis* infection, whilst being safe for use in immunocompromised populations. The major downside to subunit vaccines is their low immunogenicity, which requires that they be combined with an appropriate adjuvant and delivery system in order to make them effective. This review focuses on adjuvants and delivery systems for use in novel subunit TB vaccines, their mode of action, and likely impacts on the anti-TB immune response.

### 1.1. Subunit TB Vaccine Candidates

Subunit vaccines rely on the selection of an appropriate protective antigen. Antigen selection for TB vaccines is complex, because of the intricacy of multi-staged infection and the large size of the *M. tuberculosis* genome. The most commonly used antigens in subunit TB vaccines are conserved secreted proteins, such as ESAT6 and Ag85B, which have been shown to be immunogenic in animal models [11]. However, a multitude of other antigens, including non-secreted immune targets [12], have also been tested as vaccines [13]. One such antigen is CysD, an essential protein in the sulphur assimilation pathway of *M. tuberculosis* that is upregulated during latent infection and is highly conserved across strains, which has been utilized in our novel subunit vaccine CysVac2 [12]. CysVac2 combines CysD with Ag85B, a secreted early stage antigen, and the vaccine has been shown to be effective both as a preventative and therapeutic vaccine, including when formulated with the Advax™ polysaccharide adjuvant [14]. This illustrates the advantage of a subunit vaccine design, which allows for the targeting of different infection stages. 

### 1.2. TB Vaccine Adjuvants

**Figure 1:**
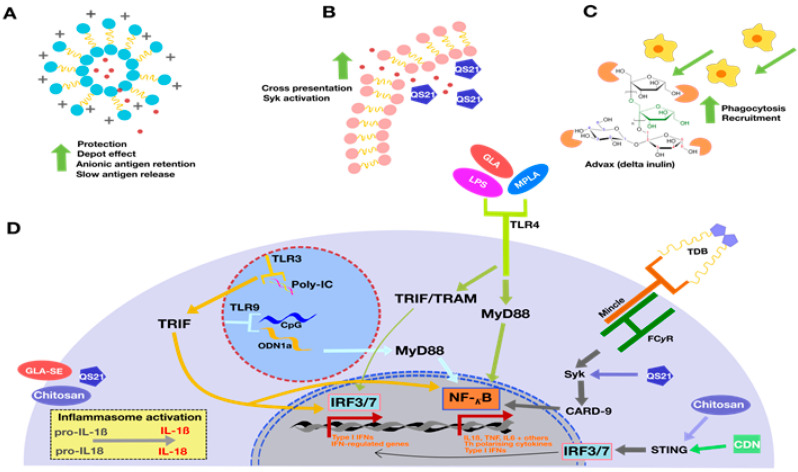
Proposed mechanism of action of adjuvants used in subunit tuberculosis vaccine candidate formulations. Many compounds exploit structural features to achieve adjuvanticity (**A**–**C**). Liposomal formulations, particularly cationic liposomes (**A**), protect and retain anionic vaccine antigens whilst creating a depot effect that potentiates slow antigen release. Adjuvant components such as QS21, found in AS01, interact with and disrupt the liposomal membranes (**B**), enhancing cross presentation to CD8^+^ T cells and inflammatory cytokine production via the Syk tyrosine kinase pathway. The novel polysaccharide adjuvant, Advax™, (**C**) potentiates phagocytosis and recruits immune cells to the site of vaccination, despite minimal inflammatory effects. Other adjuvants rely on distinct molecular pathways known to induce inflammation, such as the activation of pattern recognition receptors, both intracellular (Poly:IC (toll-like receptor (TLR)3, TLR7/8, or CpG oligonucleotides (TLR9)) or extracellular (TLR2, 3-O-desacyl-4′-monophosphoryl lipid A (MPLA; TLR4), and Mincle). Chitosan and cyclic dinucleotides (CDNs) activate the cytoplasmic DNA sensor STING.

The administration of the TB antigen alone generally fails to generate a sufficiently strong protective adaptive immune response. T-cells require secondary co-stimulatory signals, usually provided by innate immune activation and cytokines, in addition to binding the of the antigen by the T-cell receptor (TCR). Subunit vaccines require a second component, therefore, known as an adjuvant, to adequately stimulate and activate the immune response to the vaccine. Adjuvant selection is critical, as different adjuvants stimulate the immune system in different ways, some of which may not be protective, making adjuvant choice pivotal to vaccine success. The adjuvant shapes the adaptive immune response, depending on which innate immune receptors it activates (Figure 1). Thus, adjuvants may be used to generate the appropriate type of immune response needed for protection against a specific pathogen. For example, adjuvants may activate pattern recognition receptors (PRRs), such as a toll-like receptors (TLRs; e.g., nucleic acid analogues and bacterial cell wall components), nucleotide-binding oligomerisation domain (NOD)-like receptors, or retinoic acid-inducible gene-I (RIG-I)-like receptors, each of which initiate different downstream cytokine signaling [15]. However, the precise mechanisms of many adjuvants are still unclear and may not necessarily involve a specific PRR. One of the most broadly used adjuvants, alum, was believed to be reliant on antigen depot formation for its adjuvanticity [16], but also activates the inflammasome and induces the secretion of inflammatory cytokines, including interleukin (IL)-1β, which enhances dendritic cell (DC) activation [16,17]. The physiological outcome of formulating vaccines with alum is the enhancement of the antibody response in association with a major Th2-bias to the immune response.

A key focus of the TB vaccination has been to generate strong Th1 responses. Many strategies have focused on the activation of TLRs and downstream IL-12 secretion to promote Th1 polarization. This has been achieved by the use of adjuvants that bind various TLRs, such as Poly:IC (TLR3), 3-O-desacyl-4′-monophosphoryl lipid A (MPLA; TLR4), or CpG oligonucleotides (TLR9) [18,19]. A common adjuvant in many preclinical TB vaccines has been the combined formulation of dimethyldioctadecyl-ammonium (DDA) with MPLA, resulting in an effective but highly inflammatory combination not suitable for human use [20].

More recent studies have identified that a balance of Th1 and Th17 immunity may be more effective to protect against *M. tuberculosis* [21]. Notably, this balance may be altered by the route of vaccine administration, as mucosal vaccine administration steers towards Th17 responses [22,23]. However, T-cell differentiation is also influenced by the choice of adjuvant; for example, TLR4 and TLR7/8 agonists can promote Th17 responses by inducing IL-23 expression [24]. CAF01, a liposomal adjuvant formulation comprising DDA mixed with a glycolipid immunomodulator (trehalose 6,6-dibehenate, TDB), a synthetic variant of the mycobacterial cord factor, was shown to induce Th17 responses via the activation of the C-type lectin receptor, Mincle [25]. Similarly, cyclic dinucleotides generate long-lived immunity by activating the cGAS-STING (cyclic GMP-AMP synthase-stimulator of interferon genes) pathway, a strategy that is hypothesized to mimic *M. tuberculosis* intracellular infection [26]. 

The role of mucosal immunity in infection by *M. tuberculosis* may be of crucial importance to TB vaccine design, with anatomic features of the lung integral to the generation of effective immunity [13,27]. For example, M-cells (microfold cells) are a mucosa specific cell type that, in the respiratory tract, are located in the nasal associated lymphoid tissue (NALT) and inducible bronchial associated lymphoid tissue (iBALT). Their transcytosis abilities and close proximity to DCs beneath the mucosal epithelium allows M-cells to quickly transport antigens and stimulate an immune response, so they have been investigated as potential target sites for vaccine antigen administration [28,29]. Adjuvants such as polyethyleneimine and chitosan have also been used as penetration enhancers and immunostimulants for nasally administered vaccines, because of their ability to bind and cross the mucosal epithelium, with a high efficiency to access resident antigen presenting cells (APCs) [30].

## 2. Adjuvants in Clinical-Stage TB Vaccines

Whilst the clinical pipeline of TB vaccines is highly diverse, subunit vaccines seem ideal TB candidates, particularly for use in boosting BCG-induced immunity. There are four subunit vaccines aimed at the prevention of disease currently in clinical testing, and three aimed at therapeutic use [4]. In these vaccines, the adjuvants used include TLR agonists, liposomal formulations and combinations of both, thereby targeting different immune pathways and utilizing different delivery vehicles. 

### 2.1. Liposomal Formulations and Emulsions—AS01, CAF01, and GLA-SE

A strategy employed by many candidate TB vaccines is the use of liposomes and emulsions as a delivery vehicle. Liposomes are lipid-based vesicles that self-assemble through hydrophobic interaction, forming microparticles able to carry different vaccine or adjuvant formulations [20]. One of the benefits of microparticle formation is that, depending on the size of the particles, antigens will either be targeted to the lymph nodes via drainage through the lymphatic system, or will be actively transported by APCs [31]. Liposomes are effective as adjuvants, partially because after injection, loaded antigens are slowly released, with their vesicular structure protecting enclosed antigen from degradation whilst forming a depot [19,32]. Additionally, liposomes formulated to be negatively charged (cationic liposomes) aggregate, and are also able to bind positively charged antigens, enhancing this depot effect further [33,34]. Cationic liposomes have also been shown to raise the lyososomal pH following DC antigen internalization, reducing antigenic degradation and enhancing the level of cross presentation to CD8^+^ T-cells via major histocompatibility complex (MHC)-I [35,36,37]. However, liposomal and emulsion formulations are often associated with local site reactogenicity, and many formulations have had to be revised to improve their safety profile prior to use in humans [38,39].

One of the most advanced subunit vaccines, M72:AS01, was recently shown to have 54% efficacy in HIV-negative individuals with latent TB when administered intramuscularly (M72:AS01_E_) [40]. The adjuvant in this vaccine, AS01, consists of a mixture of the TLR4 ligand, MPLA, together with the saponin fraction QS21 in a liposomal formulation [41]. MPLA is a derivative of the lipopolysaccharide from *Salmonella minnesota* modified to reduce its toxicity and is commonly used in adjuvant formulations for its ability to bind TLR4 and induce NF-κB activation [42,43]. QS21 is a mixture of two isomeric triterpene glycosides, arabinose (QS-21A) and xylose (QS21X), isolated from the tree *Quillaja saponaria* Molina [44]. Saponins are amphiphilic glycosides, of which the most commonly researched are isolated from *Q. saponaria*, namely Quil A, and its derivative, QS-21 [45]. The mechanism of action of AS01 in M72:AS01 is proposed to be via rapid interferin (IFN)-γ production by resident natural killer (NK) cells and CD8^+^ T cells in the draining lymph nodes promoting strong cellular Th1 responses [46], a theory supported by the robust Th1 and IFN-γ responses observed during human trials of the vaccine [40,47,48].

Similarly to M72:AS01, the adjuvant used in H1:CAF01, a phase I candidate TB vaccine, is a liposomal formulation consisting of DDA and TDB [49]. DDA is a synthetic amphiphilic lipid capable of self-assembling into vesicles. Alone, DDA is unstable and will form aggregates, however TDB incorporates into DDA bilayers and stabilizes the liposomes [50]. TDB is also highly immunostimulatory, activating Mincle. Upon the recognition of TDB, Mincle interacts with Fc receptor common γ-chain (FcRγ) inducing intracellular signaling via Syk, causing CARD9 dependent NF-κB activation and downstream proinflammatory cytokine production [25,51]. Adjunctive to NF-κB activation, CAF01 also relies on Mincle-dependent IL1 production and the subsequent MyD88 signaling to generate Th1/Th17 polarized responses [52]. In preclinical trials of the H1:CAF01 vaccine, the CAF01 adjuvant generated significant parenchymal IFN-γ producing T-cells, and induced Th17 dependent memory and protection when the vaccine was used either pre- or post-*M. tuberculosis* exposure [53,54,55]. In humans, H1:CAF01 induced multi-functional antigen specific T-cell responses lasting up to three years post vaccination [49]. 

The synthetic TLR4 agonist, glucopyranosyl lipid adjuvant in squalene oil in water emulsion (GLA-SE), is the adjuvant used in the ID93/GLA-SE vaccine currently in phase II trials [56]. GLA is a synthetic TLR4 agonist that promotes polyfunctional responses via MyD88 and TIR-domain-containing adapter-inducing interferon-β (TRIF)-dependent activation [57]. The delivery vehicle of GLA-SE is crucial to its adjuvanticity, with a recent study demonstrating that GLA alone promotes IgG2 responses, as does squalene emulsion alone, whereas GLA-SE combined induces a Th1 response [58]. Squalene oil in water emulsions are found in multiple adjuvants including MF59, which is licensed for use with influenza vaccines for the elderly, although their mechanism of action remains unclear [38].

### 2.2. Other Adjuvants in Clinical Trials: IC31 and GamTBVac

Whilst liposomes and oil-in-water emulsions are a popular adjuvant strategy for inducing cell-mediated immunity, IC31, an adjuvant employed in two TB vaccines currently undergoing clinical trials, utilizes a cationic peptide as a delivery vehicle. IC31 is used both in post exposure vaccine H56:IC31 and the preventative vaccine H4:IC31, the latter in a recent phase II trial found to have 30% efficacy in the prevention of *M. tuberculosis* infection [59]. IC31 consists of the antimicrobial peptide KLKL_5_KLK (KLK) combined with a single stranded oligonucleotide, ODN1a, which is thought to bind TLR9, thereby activating the MyD88 pathway and IL-12 production by APCs [19,60]. KLK is also immunostimulatory and is hypothesized to allow for translocation into cells without cell membrane permeabilization, thus making access to intracellular TLRs more efficient [61]. Counterintuitively, however, KLK has also been investigated for its anti-inflammatory potential whereby it reduces nitric oxide, IL-1β, and TNF production caused by LPS exposure [62]. In a vaccine setting, KLK when administered alone with antigen induced a Th2 type immune response, however, when combined with ODN1a, it induced a stronger Th1 and Th2 immunity [60,63].

Finally, GamTBVac, a subunit vaccine under phase I clinical development, utilizes a dextran and CpG adjuvant along with an antigen fusion protein containing a dextran binding domain [64]. Dextran has a history of medical usage and has the advantage of being classified as “generally recognized as safe” (GRAS) by the FDA. In an adjuvant setting, dextran may induce inflammation by interacting with the DC-SIGN family receptors, the mannose receptor, and langerin, all of which may induce innate immune activation [65].

## 3. Novel TB Vaccine Adjuvants

While only a few adjuvants have so far been used in TB clinical trials, there are a vast number of delivery vehicles and adjuvant strategies in preclinical research (see Table 1). The majority of these adjuvants may be broadly divided into three groups, namely: nano- or micro-particulate, delivery system-based and plant or microbial derived. Many strategies are specifically aimed at activating PPRs with downstream inflammatory effects and immune cell recruitment, while others potentiate vaccine protection through currently unknown mechanisms. There is a growing body of research exploring the adjuvants suitable for pulmonary or intranasal administration, given the potential advantages of mucosal delivered TB vaccines [13,66,67]. In this setting, adjuvants must avoid damaging the sensitive lung tissue. It is also increasingly obvious that the magnitude of cytokine release following the re-stimulation of antigen specific T cells may be an inaccurate measure of vaccine protectiveness against TB infection [68,69,70]. In response to this paradigm shift, there has been increasing interest in the design of adjuvants with less inflammatory mechanisms of action, which should thereby be safer for pulmonary administration.

### 3.1. Nanoparticles and Microparticles: Travelling Different Immune Pathways to Reach the Lymph Node

Both nanoparticles and microparticles, including the use of liposomes, are very popular strategies because of their ability to specifically target cell populations based on the size, surface chemistry, and administration site of these particles [93]. A common polymer that is used is poly(DL-lactic-co-glycolic acid; PLGA) which, like dextran, has a long history of medical usage prior to its adjuvant applications, and has already been approved for parenteral use for sustained drug delivery by the FDA [94,95]. PLGA can be manufactured into either nanoparticles (<1 µm) or microparticles (>1 µm), depending on many factors, including the concentration of surfactants and polymers, as well as the homogenization speed [95]. There has been much interest in the use of PLGA microparticles for the delivery of anti-TB vaccines and treatments [88,89,95,96,97,98]. The mycobacterial Hsp65 protein along with KLK encapsulated in PLGA microspheres of ~7 µm in size was highly protective when administered intramuscularly as a single dose [97]. Surprisingly, this vaccine induced high levels of IL-10 and lower levels of IFN-γ compared to a similar vaccine where KLK was replaced with CpG, indicating, as in other studies, that high inflammatory cytokine readouts and particularly IFN-γ are not necessarily correlates of TB vaccine efficacy. However, this study did not characterize the role of Th17 polarized CD4^+^ T cells, which have been recently shown to be important in the generation of resident immune memory [53]. Whilst PLGA microspheres may induce robust antibody and Th17 responses, vaccines incorporating PLGA particles do not appear to improve the efficacy of TB vaccine formulations utilizing DDA and TDB liposomes (CAF01) [88,89]. Thus, the effectiveness of PLGA particles as TB vaccine carriers remains to be confirmed. 

Other vaccine strategies that have been utilized against TB include the use of particles suitable for pulmonary delivery. Often, the micro- or nano-particles intended for use in the mucosa are of biological origin, because of their ability to induce IgA antibody responses, enhance movement through the mucosa, heightened mucoadhesion and resistance to enzymatic degradation [73,92,99]. Reljic et al. demonstrated that inert *Bacillus subtilis* spores promote DC maturation, recruit NK cells to the lungs and activate the NK-κB pathway [100]. Based on these observations, they tested mice spores approximately 1 µm in size coated with a TB antigen administered either intranasally or subcutaneously as a BCG booster, whereby they observed a protective response against *M. tuberculosis*, which was associated with the generation of IFN-γ expressing CD4^+^ T cells [73]. Derivatives of chitosan, a linear polysaccharide that forms part of the exoskeleton of shellfish, have also been utilized to create immunostimulatory carrier vehicles for antigens [78]. Chitosan derivatives enabled DC activation and Th1/Th17 polarization via type-I IFN production with their effects on DC activation abrogated in STING knockout mice. It is hypothesized that upon internalization, chitosan derivatives induce mitochondrial stress and reactive oxygen species production, causing the release of mitochondrial DNA that may trigger the cGAS-STING pathway resulting in a Th1/Th17 response [26,77]. Thus, natural polymers and particles provide a vast array of sources for adjuvant particulate delivery systems. 

### 3.2. Adjuvants Derived from Nature: Plant and Microbial

Many particulate adjuvants are derived from natural sources. Polysaccharides are gaining attention as adjuvants because of their biocompatibility, biodegradability, and innate immune modulation capacities [101]. Natural polysaccharides have the ability to activate many immune cells, including macrophages and T- and B-lymphocytes, and subsequently cause the downstream expression of chemokines and cytokines [101]. Advax™ is a novel plant-derived polysaccharide that, when formulated into delta inulin particles, has been shown to enhance vaccine immunity against many diseases. Advax™ is made from inulin particles isolated from the roots of *Compositae* plants [102]. Inulin has long been used in medicine to measure glomerular filtration; however it was also observed that insoluble fractions of inulin were capable of activating complement [103], leading to the identification of an alternative complement pathway [104]. In its delta isoform, inulin forms cationic particles of ~2 µm in diameter that remain highly insoluble in water at 50 °C, and when administered subcutaneously or intramuscularly with the CysVac2 antigen induced robust multifunctional CD4^+^ T cell responses and protection against *M. tuberculosis* challenge [14]. Amongst its immunological effects, Advax™ induces a strong chemotactic effect, resulting in the recruitment of leukocytes to the site of vaccination, and stimulates a broad-based immune response to co-administered antigens, including both humoral and Th1, Th2, and Th17 T-cell responses [14,105,106]. 

An advantage of Advax™ as a candidate for the TB vaccine clinical development pipeline is its demonstrated high tolerability and safety in previous human trials, when included in vaccines against influenza, hepatitis B, and allergy [107,108,109,110]. As a part of an influenza vaccine assessed in phase I trials, Advax™ provided antigen dose-sparing with low reactogenicity, a positive outcome repeated in all of the human trials in which it has been tested [107,108,110]. Furthermore, in mouse models, Advax™ has been shown to be a safe and effective adjuvant for pulmonary administration, enhancing the protection of the mice from a lethal influenza challenge via the enhancement of both humoral and cell mediated immunity [111]. Thus, the comprehensive engagement of multifaceted immune pathways combined with its demonstrated safety in humans, makes Advax™ a strong adjuvant candidate for use in TB vaccines entering clinical trials. 

An adjuvant system comprised partially of plant derived factors is the ISCOM, or immune stimulating complex, a group of adjuvants that consist of saponin, cholesterol, and phospholipid, organized into cage-like structures 40–50 nm in diameter. ISCOMs recruit NK cells, lymphocytes, DC, and granulocytes to the draining lymph node following administration, and generate a Th1/Th2 response [82]. It has been suggested that the action of ISCOMs is TLR independent, but MyD88 dependent [82,112]. Andersen et al. tested an intranasal BCG booster vaccine, in which they utilized ISCOMs in combination with CTA-1/DD, a cholera toxin derived fusion protein with adenylate cyclase activity leading to cAMP accumulation [83,113,114]. The response induced by this vaccine was both humoral- and cell-mediated, with a high IFN-γ production both systemically and locally.

## 4. Future Strategies and Developments

The design of a more effective vaccine against TB will require filling of the “knowledge gaps” regarding true correlates of protective immunity to *M. tuberculosis* infection. Currently, there are no completely reliable correlates making adjuvant selection for novel vaccine candidates largely empiric. A further challenge to the understanding of TB vaccine efficacy is the time it takes for *M. tuberculosis* infection to progress to symptomatic stages, the ability of *M. tuberculosis* for latent infection, and the lack of efficient and accurate diagnostic tools easily translatable to rural and developing settings [115]. The generation of multi-functional cytokine secreting Th1 cells expressing IFN-γ, IL-2, and TNF were previously considered the critical subset that correlated with protective immunity, thus the induction of these multi-functional CD4^+^ T cells has been the goal of most TB vaccine adjuvants [116]. However, recent studies have challenged this notion, as good multi-functional T-cell generation by various vaccines did not lead to significantly better protection against TB [68]. Furthermore, excessive Th1 differentiation may even inhibit the development of T cell subsets that actually mediate protection [8,117]. Excessive antigen doses in post-exposure vaccines reduced the longevity of protection against TB in mice, and in a pre-exposure setting, a 1000-fold lower antigen dose gave better long term protection [118]. 

There has been recent renewed interest in mucosal vaccines, and, in particular, pulmonary vaccination. The reasoning is that immunity should be generated at the site of infection for maximal effectiveness [13,119,120]. Th17 cells induced by mucosal vaccination develop into long-term resident memory cells, and the efficacy of many mucosal vaccines surpasses the efficacy of the same vaccines administered parenterally [66,67]. There are currently no adjuvants specifically designed for pulmonary administration, but such adjuvants would need to have low inflammatory capacity and elicit strong mucosal immunity [30,53]. Advax™ adjuvant fits these criteria, given its high safety profile and lack of inflammatory reactogenicity, and its ability to induce a broad T cell response including Th1, Th2, and Th17 CD4+ subtypes, together with memory CD8 T-cells.

We speculate that the development of an effective TB vaccine to meet the ambitious goals set out in the World Health Organization “End TB” strategy will require a radical change in thinking, away from traditionally accepted immune correlates of protection, parenteral administration, and traditional highly inflammatory adjuvants. A move towards mucosal administration and avoiding excessive immune stimulation and inflammation will necessitate the development of adjuvants that are immune-stimulatory without being inflammatory. Advax™ is a highly effective adjuvant, despite its benign sugar composition, and in partnership with CysVac2 antigen, may be effective when given either parenterally or though the intrapulmonary route.

## Figures and Tables

**Table 1:** Summary of adjuvant strategies in human TB vaccine clinical trials or in preclinical animal testing. MPLA:3-O-desacyl-4′-monophosphoryl lipid A; DDA:dimethyldioctadecyl-ammonium; TDB:trehalose 6,6-dibehenate; GLA:glucopyranosyl lipid adjuvant; KLK:KLKL_5_KLK; TLR:toll-like receptor.

Adjuvant/Delivery System	Components	Antigen	Proposed Mechanism of Action	Immune Readout	Testing Status	References
Advax	Delta inulin particles	Ag85B, CysD (CysVac2)	Enhanced phagocytosis, immune cell recruitment, low reactogenicity	Th1, Th17	Preclinical	[12,14]
AS01	MPLA and QS21	Mtb32, Mtb 39 (M72)	TLR4 activation (MPLA), liposomal disruption and Syk activation, CD2 activation on T-cells, NLRP3 inflammasome (QS21)	Th1	Phase IIb (54% efficacy)	[40,71,72]
*B. subtilis* spores		MPT64; Acr-Ag85B	Mucoadhesive, resistant to enzymatic degradation, suitable for mucosal administration	Th1, IgA, low Th17	Preclinical	[73,74]
CAF01	DDA and TDB	Ag85B, ESAT-6 (H1)	TDB activates Mincle, MyD88-dependent Th1/Th17 polarising cytokines. DDA forms cationic liposomes that are stabilised by TDB.	Th1, Th17	Phase I	[49,52,75,76]
Chitosan and derivatives		Ag85B, ESAT-6 (H1)	Activates cGAS-STING pathway, mucoadhesive and mucosal epithelial penetration properties, suitable for mucosal administration	Th1, low Th17	Preclinical	[77,78]
Cyclic dinucleotides	Synthetic dinucleotide analogue of cyclic diguanylate	Ag85B, ESAT-6, Rv1733c, Rv2626c, RpfD (5Ag)	STING activation (IRF-3 type I IFN production, NFkB, STAT-6 chemokine expression)	Th17, low Th1	Preclinical	[26]
Dextran		Ag85A, ESAT-6-CFP10	Activates DC-SIGN receptor family, mannose receptor, langerin	Th1/Th2	Phase I	[64,65]
GLA-SE	GLA in squalene emulsion	Rv2608, Rv3619, Rv3620, Rv18183 (ID93)	GLA is a synthetic TLR4 agonist, in squalene in water emulsion activates NLRP3 inflammasome	Th1	Phase IIa	[56,57,58,79]
IC31	KLK and ODN1a	Ag85V, ESAT-6 (H1); Ag85B, ESAT-6 and Rv2660c (H56) and Ag 85B, TB10.4 (H4)	ODN1a binds TLR9, KLK forms aggregates with ODN1a and enhances translocation into cells	Th1	Phase IIa (H56:IC31; 30.5% efficacy)	[46,59,80,81]
ISCOMs	Immune stimulatory complexes (saponin, cholesterol and phospholipid)	Ag85B, ESAT-6 (H1); Ag85A	TLR independent, may be inflammasome mediated (under investigation)	Th1/Th2	Preclinical	[82,83,84]
Lipokel	PamCys2 and 3NTA	Culp 1, Culp 6	PamCys2 is a TLR2 ligand and 3NTA is a chelating entity that allows antigen binding	Th1	Phase I	[85]
Nanoemulsion	Soybean oil phase mixed into aqueous phase	ESAT-6, Ag85B	Mucoadhesive, highly tolerated, suitable for mucosal administration	Th17, Th1	Preclinical	[86,87]
PLGA (poly(lactic-co-glycolic acid))	Microsphere delivery system	Ag85B, ESAT-6 (H1); MPT83	Antigen protection, depot formation, controlled release, enhanced phagocytosis, biodegradable, suitable for mucosal administration	Th1, Th17	Preclinical	[88,89]
PolyI:C	dsRNA	BCG; Ag85B, HspX	TLR3 agonist	Th1, Th2	Preclinical	[90,91]
Yellow carnauba wax nanoparticles	Incorporated with heparin-binding hemagglutinin adhesion (HBHA) protein	Ag85B	Enhanced adherence to alveolar epithelium (HBHA), highly tolerated (particles), suitable for mucosal administration	Th1	Preclinical	[92]
