# Correction: Stewart et al. Adjuvant Strategies for More Effective Tuberculosis Vaccine Immunity. *Microorganisms* 2019, *7*, 255

**DOI:** 10.3390/microorganisms10040757

**Published:** 2022-03-31

**Authors:** Erica Stewart, James A Triccas, Nikolai Petrovsky

**Affiliations:** 1 Discipline of Infectious Diseases and Immunology, Central Clinical School, Faculty of Medicine and Health, The University of Sydney, Camperdown, NSW 2006, Australia; erica.stewart@sydney.edu.au (E.S.); jamie.triccas@sydney.edu.au (J.A.T.); 2 Charles Perkins Centre, The University of Sydney, Camperdown, NSW 2006, Australia; 3 Vaxine Pty Ltd., Adelaide, SA 5042, Australia; 4 Department of Endocrinology, Flinders University, Adelaide, SA 5042, Australia

The authors wish to make the following corrections to this paper [1]:

AS01_E_ was mistakenly referred to as in emulsion form, as below:

One of the most advanced subunit vaccines, M72:AS01, was recently shown to have 54% efficacy in HIV-negative individuals with latent TB when administered intramuscularly in emulsion form (denoted M72:AS01_E_) [40]. The adjuvant in this vaccine, AS01, consists of a mixture of the TLR4 ligand, MPLA, together with the saponin fraction QS21 in a liposomal or emulsion formulation [41].

This should be changed to the correct version, as follows:

One of the most advanced subunit vaccines, M72:AS01, was recently shown to have 54% efficacy in HIV-negative individuals with latent TB when administered intramuscularly (M72:AS01_E_) [40]. The adjuvant in this vaccine, AS01, consists of a mixture of the TLR4 ligand, MPLA, together with the saponin fraction QS21 in a liposomal formulation [41].

The authors would like to apologize for any inconvenience caused to the readers by these changes and state that the scientific conclusions are unaffected. The original publication has also been updated.
